# Pain experience of lung cancer patients during home recovery after surgery: A qualitative descriptive study

**DOI:** 10.1002/cam4.6616

**Published:** 2023-10-03

**Authors:** Cheng Lei, Ruoyan Gong, Jiayuan Zhang, Kejimu Sunzi, Na Xu, Qiuling Shi

**Affiliations:** ^1^ School of Public Health Chongqing Medical University Chongqing China; ^2^ Nursing Department People's Hospital of Deyang City Sichuan China; ^3^ Department of Thoracic Surgery Sichuan Cancer Hospital & Institute Sichuan China; ^4^ State Key Laboratory of Ultrasound in Medicine and Engineering Chongqing Medical University Chongqing China

**Keywords:** lung cancer, pain experience, postoperative, post‐recovery, qualitative research

## Abstract

**Background:**

Patients with lung cancer surgery often suffer pain after discharge. However, there is limited evidence to describe the pain experience from the qualitative investigation. This study was designed to describe the pain experience of lung cancer patients during home recovery after surgery and to provide evidence for developing pain management strategies.

**Methods:**

This qualitative descriptive study recruited 35 patients who had received lung resection and were discharged by purposive sampling from May to July 2022. Mobile phone interviews were conducted to collect views on patients' pain experience after discharge. The interviews were audio‐recorded and converted verbatim into standard text, and the data were iteratively thematic analyzed.

**Results:**

A thematic framework was identified for three themes: perception and impact of pain, coping styles for pain, and unmet needs for pain. Whether short or long‐term after discharge, patients complain that they suffer from different degrees and types of pain, causing them a “double burden” physically and mentally. The lack of knowledge about pain may lead them to adopt very different coping styles and desire professional continuous pain management.

**Conclusions:**

This study may help clarify the status of pain problems that patients face after lung cancer surgery and provides multiple dimensions of pain assessment and management objectives for quantitative research. We call on medical staff to pay attention to patients' perspectives and related needs after discharge and formulate targeted management strategies to reduce pain and improve their quality of life.

## 1 BACKGROUND

In 2020, 2.2 million new cases of lung cancer and 1.8 million lung cancer‐related deaths were reported globally.^1^ China faces challenges in preventing and treating lung cancer, with 871,000 new cases and 767,000 deaths in 2022, accounting for 18.1% and 23.9% of all new cases and fatalities.^2^, ^3^

Surgical intervention is a guideline‐recommended approach to lung cancer treatment; timely and comprehensive surgical intervention can extend a patient's lifespan.^4^, ^5^ Patients will have acute systemic inflammation, neuroendocrine, and metabolic stress response after surgery, usually characterized by a series of nonspecific unpleasant symptoms, which may lead to considerable dysfunction, among which pain is one of the most prevalent.^6^, ^7^, ^8^ Pain, the fifth vital sign, is “an unpleasant sensory and emotional experience associated with, or resembling that associated with actual or potential tissue damage”.^9^ Previous research corroborates that pain is the most frequently occurring and perceptibly prominent postoperative discomfort from the perspective of both patients and healthcare providers.^10^ A systematic review pointed out that the incidence of chronic pain after video‐assisted thoracic surgery is 35.3%, with 10.5% of patients reporting moderate or severe pain.^11^ Compared to baseline, pain perception escalates 1–3 months following surgery; it gradually decreases over time, potentially returning to baseline levels around six months.^12^, ^13^ However, a subset of patients may develop long‐term chronic pain. A decade‐long retrospective study confirmed that 17.4% of patients were diagnosed with postoperative chronic pain 36 months after lung surgery.^14^

Pain has a more pronounced impact on the quality of life post‐surgery than the patient's understanding of the treatment plan, participation in decision‐making, or trust in their medical practitioners.^15^, ^16^ In addition, there is a connection between pain symptoms and the beginning of anxiety episodes. If pain after surgery is not controlled well, it could develop symptoms associated with post‐traumatic stress disorder (PTSD) and affect the patient's psychological well‐being.^17^ Research indicates that up to half of patients who have undergone lung cancer surgery have reported experiencing PTSD.^18^ Consequently, postoperative pain, directly and indirectly, affects the patient's health‐related quality of life.

Interventions have been established for hospitalized patients' pain, and their effectiveness has been proven.^19^, ^20^, ^21^ However, with the shortening of hospital stays, the home recovery of lung cancer patients necessitates long‐term comprehensive management and attention.^14^, ^22^ The current study focuses on pain assessment of clinical indicators and physical aspects and lacks focus on individualized patient experience. Knowledge of their pain experience remains very limited. Thus Understanding patient feelings and needs for pain holds significant implications for formulating a holistic postoperative recovery management plan.

This study aimed to depict the pain experience of lung cancer patients recuperating at home post‐surgery, providing a basis for formulating targeted measures for long‐term pain management.

## 2 METHODS

### 2.1 Study design

We utilized a qualitative descriptive design based on a naturalistic inquiry to delineate lung cancer patients' pain experiences during their home recovery after surgery, providing a foundation for formulating targeted pain management measures. The qualitative descriptive design was selected as it is extensively employed in research on health care and care‐related phenomena. It focuses on the feelings or experiences of specific individuals in particular phenomena or events, deriving insights into relatively unknown phenomena, and provides support when directly describing phenomena or seeking information to develop and improve questionnaires or interventions.^23^ The research is guided by the Standards for Reporting Qualitative Research (SRQR) checklist, which aids in enhancing transparency and maintaining an audit trail of the qualitative study process.^24^

### 2.2 Participants

From May to July 2022, patients who had received lung resection and were discharged from the thoracic surgery department of a tertiary cancer hospital in Sichuan, China, were recruited by a purposive sampling method. They all participated in the CN‐PRO‐Lung 3 (Registration: ChiCTR2000033016.), a multicenter, prospective, observational cohort study conducted in China.^25^ The recruitment criteria were: (i) at least 18 years old; (ii) diagnosed with lung cancer through imaging and pathological examination and had undergone pulmonary tumor resection; (iii) had experienced pain during the postoperative recovery period (this criterion was based on patient‐reported pain symptoms, with the standard being a report of at least three instances of pain rating higher than 3 on a 0–10 scale); (iv) had signed an informed consent form and cooperated with the interview process. Analyzing the text obtained from interviews is an iterative process. The sample size was determined by reaching “thematic saturation.”.^26^ We interviewed two more individuals on this basis after interviewing 33 individuals and still have not identified any new themes. In the end, 35 patients were interviewed.

### 2.3 Data collection

Before the interview, we obtained a list of potential participants from the CN‐PRO‐Lung 3 database. We exported their sociodemographic and clinical features in advance. Then, potential participants were contacted via mobile phone. Once they accepted the invitation, the interview was conducted immediately, or a time was scheduled for a phone interview according to their schedules. Each participant was interviewed for at least 15 min. All interviews were conducted by the first author (CL), a registered nurse pursuing a doctoral degree, who had received comprehensive training in qualitative research methodology, held experience in multiple qualitative research studies, and enclosed many years of clinical nursing practice, mastering communication skills with patients. A paper notebook was used to record important content during the interview process (such as the patient's tone, repetition of a particular description, and the interviewer's feelings and thoughts about the current situation). A semi‐structured interview outline was developed to assist in telephone interviews (S1 in the Supplementary Material). The process was recorded with the informed consent of the participants, and the audio files were transcribed verbatim into text documents within 48 hours after the interview.

### 2.4 Data analysis

The data analysis began as soon as the first patient's interview text was obtained and was then carried out in tandem with data collection in an ongoing comparison and iterative process. Two researchers (CL and RG) conducted independent coding and extracting using thematic analysis involving six steps (S2 in the Supplementary Material).^27^ NVivo 12 (NVivo qualitative data analysis software; QSR International Pty Ltd., Version 12, 2018) was used to assist with data analysis. To illustrate the formation of themes from codes, we employed RAWGraphs, an online platform (https://www.rawgraphs.io/) that offers a free and open‐source tool for data visualization, to craft the alluvial diagram.

### 2.5 Trustworthiness

We implemented various quality measures to ensure the reliability of our research results: (1) By employing maximum variation sampling, we intentionally selected participants that represented different categories of age, education level, socio‐economic status, and gender, allowing us to capture more diverse themes. (2) Audio files were transcribed into standardized text by a professional third‐party service provider (https://www.iflyrec.com/). The transcribed text was separately checked by two experienced researchers (CL and RG) against the interview notes to ensure the text's authenticity and maintain the “restoration of the situation” as much as possible. (3) Two authors (CL and RG) independently analyzed the data, evaluated the formed codes consistently, and resolved differences in interpretation through discussion. As such, the risk of imposing personal views on the analysis was minimized. (4) To enhance the confirmability of our research results and the participation of patients and the public, we sent a preliminary report summarizing the research findings and conclusions to all 35 patients, as well as two thoracic surgery specialists and two thoracic surgery specialist nurses, to verify their reliability. (5) As all participants were conversing in Chinese, after forming the final English version of the theme description, a bilingual researcher (QS, who obtained a medical doctorate in China and has 17 years of clinical research experience at the MD Anderson Cancer Center in the United States) validated the accuracy of the translated text.

### 2.6 Ethical considerations

This study was conducted in strict accordance with the Declaration of Helsinki. It received ethical approval from the Medical Ethics Committee of the Sichuan Cancer Hospital & Institute (Approval Number: SCCHEC‐02‐2018‐043). All interviewees participated in this study voluntarily and could withdraw or retract their data anytime. All participants received a research information leaflet and signed an electronic‐based informed consent form.

## 3 RESULTS

A total of 35 patients were interviewed, with an average interview duration of 18 min (ranging from 15 to 32 min. The group included 21 females, with an average age of 54 (ranging from 28 to 72). All of them had undergone video‐assisted thoracoscopic surgery (Table 1).

**TABLE 1:** Characteristics of study participants (*n* = 35).

Characteristic	*n*	%
Age, mean (SD),years	54(11.9)	–
Gender		
Male	14	40.00
Female	21	60.00
Education		
College and above	8	22.86
High school and middle school	22	62.86
Primary school or below	5	14.29
Stages		
IA1	18	51.43
IA2	10	28.57
IB	4	11.43
IIA	2	5.71
IIIA	1	2.86
Resection mode		
Lobectomy	20	57.14
Segmentectomy	15	42.86
Post‐discharge time (±3 days)		
2 weeks	6	17.14
1 month	12	34.29
3 months	10	28.57
6 months	5	14.29
12 months	2	5.71
Times of pain score greater than 3		
3–5 times	12	34.29
5–7 times	16	45.71
8 times and above	7	20.00

**FIGURE 1:**
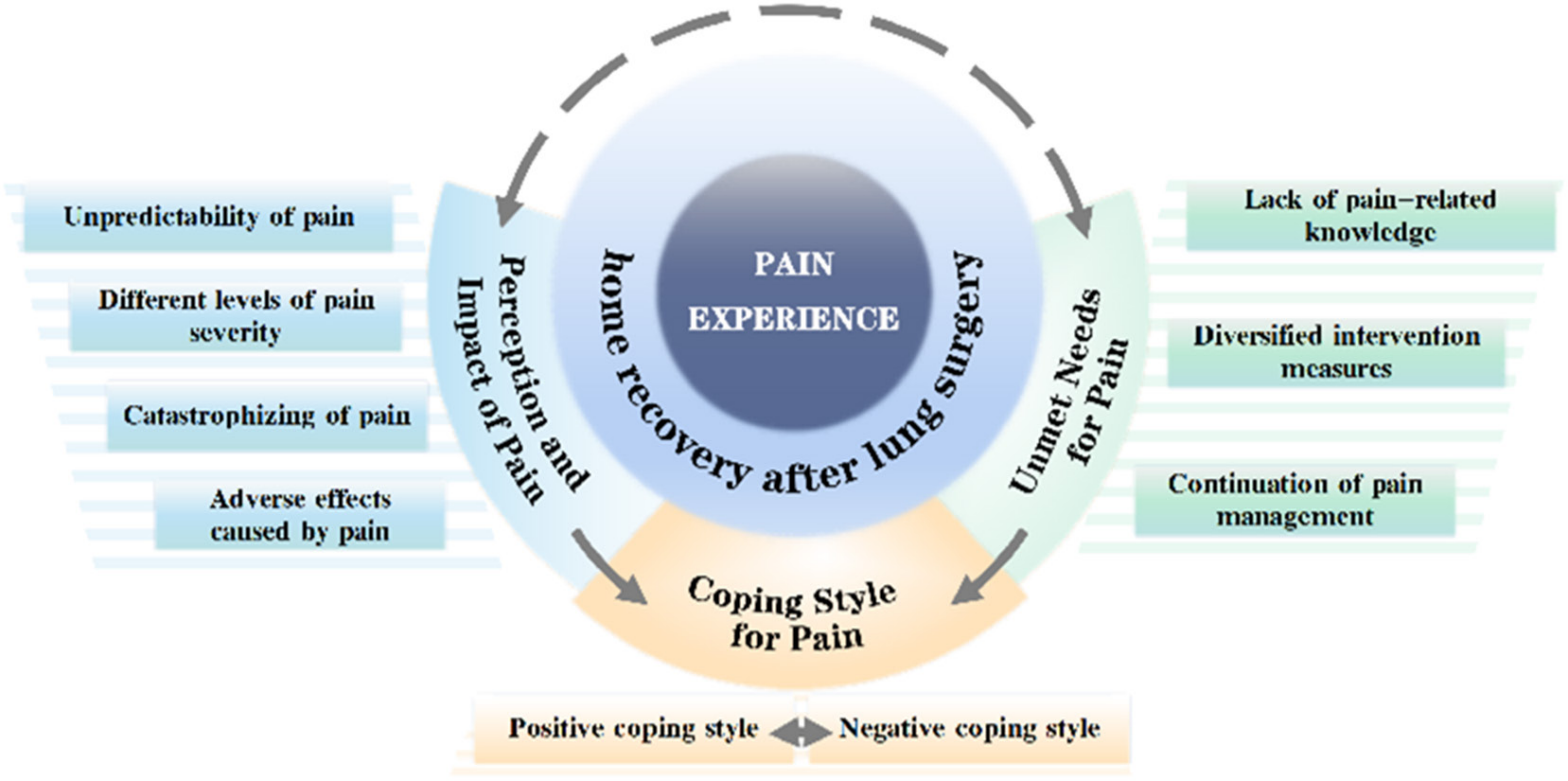
Thematic map of pain experience during home recovery after lung cancer surgery.

**FIGURE 2:**
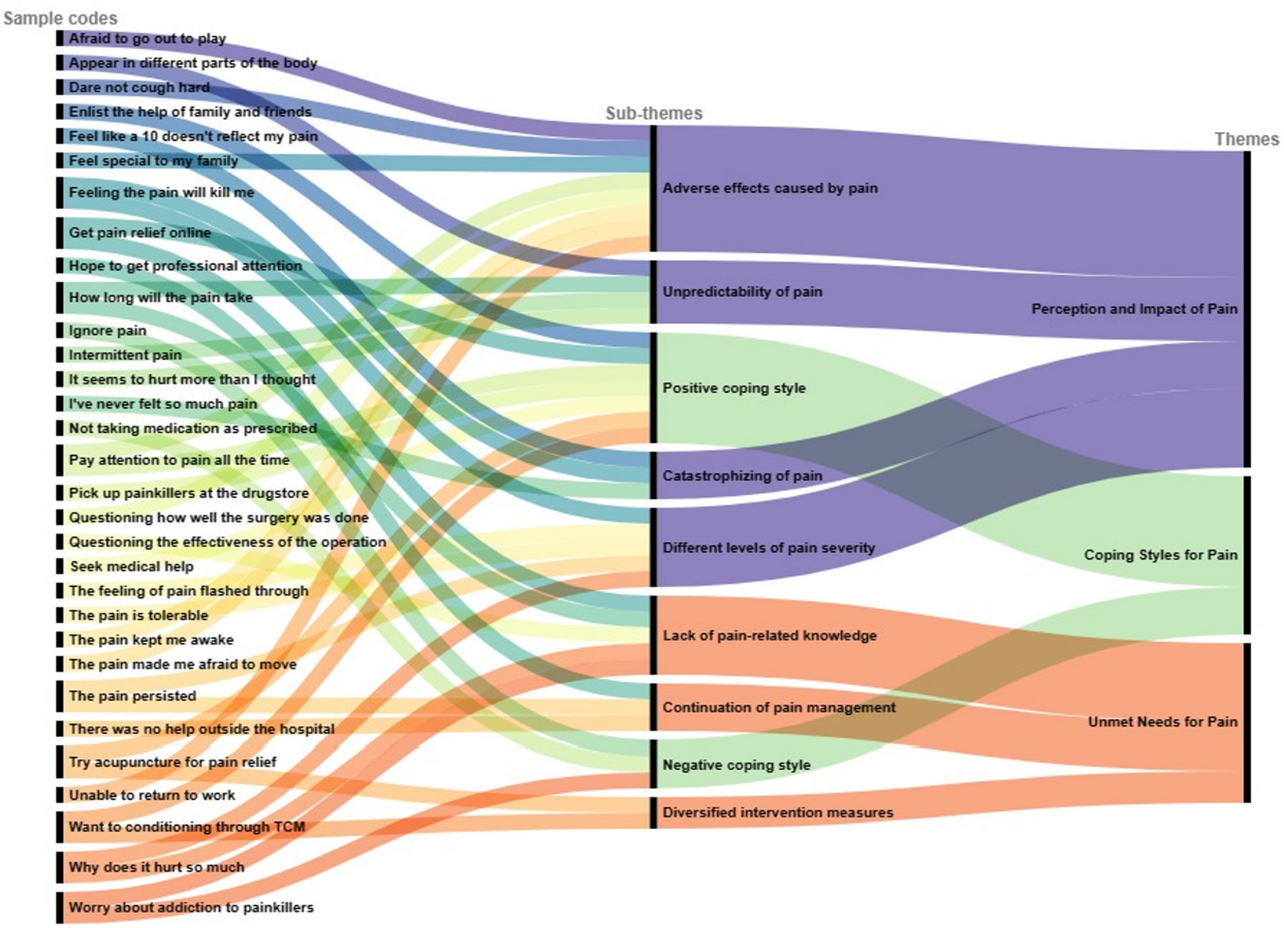
Alluvial diagram of sample codes and their subordination to each theme.

We identified three major themes and nine sub‐themes, as shown in Table 2 (S3 in the Supplementary Material Presents in more detail). A thematic map was used to display their interconnections (Figure 1), and the process from codes to themes formation was presented via an alluvial diagram (Figure 2).

**TABLE 2:** Three major themes and nine sub‐themes.

Themes	Sub‐themes
Perception and impact of pain	Unpredictability of pain
	Different levels of pain severity
	Catastrophizing of pain
	Adverse effects caused by pain
Coping styles for pain	Positive coping style
	Negative coping style
Unmet needs for pain	Lack of pain‐related knowledge
	Diversified intervention measures
	Continuation of pain management

### 3.1 Perception and impact of pain

All participants (100%) mentioned different degrees of perception of pain symptoms, and the occurrence of pain is unpredictable. Seven participants (20%) even had an overly exaggerated, catastrophic perception of pain, which may lead to more adverse effects in addition to the pain.

#### 3.1.1 Unpredictability of pain

The unpredictability of pain, especially in the postoperative environment, was reported by all participants (100%) whether in the early or medium to long‐term period after discharge. The location of the pain varies as well.

*“*I have been discharged for 2 weeks, and the wound is still excruciating. How long will it take to end?” (Participant 3, male, 57‐year‐old)

'It has been almost 2 months since my surgery, and suddenly yesterday afternoon, I started feeling a sharp pain in my chest, like a needle prick, which lasted through the night.' (Participant 12, female, 65‐year‐old)

'Last week, I suddenly felt a pulling pain in my back. It took half a day to relieve a bit, but the painkillers seemed ineffective.” (Participant 13, female, 55‐year‐old)

Twenty‐two participants (66.7%) also mentioned that the pain would persist and repeatedly at different times.

*“*When I was just discharged, the pain was severe. It was not until around a month later that it started to ease. However, recently, there have been bouts of pain from time to time. It has been almost a year, and I cannot figure out why it still hurts!” (Participant 23, male, 62‐year‐old)

Sixteen participants (45.7%) thought pain is often worse than anticipated, which can add extra stress and anxiety to the them.

“Oh my, I did not expect this. Since I had my lobectomy, the pain has been unbearable. It is much worse than I originally thought. I feel so uncomfortable every day. I hope this situation improves soon.” (Participant 8, female, 52‐year‐old).

Twenty‐four participants (68.6%) reported that the pain occurred at the surgical incision,

*“*…especially at the surgical incision, it is still very painful…”.(Participant 7, male, 55‐year‐old)

There were also other parts of the body where the pain was felt, such as,

*“*I feel a pulling pain in the shoulder area of the surgery…' (Participant 15, female, 48‐year‐old)

'I have been home for 2 weeks now, and there is still a pain in my chest.' (Participant 23, male, 52‐year‐old)

'There were a few times when my arms hurt so much I could not lift it. Is this normal?” (Participant 18, female, 48‐year‐old)

#### 3.1.2 Different levels of pain severity

Due to differences in genetics, environment, personal experiences, and psychosocial factors, the perception of pain may vary significantly between individuals. Patients had different experiences regarding the severity of their pain symptoms, with 28 participants (80%) indicating that the pain is within a tolerable range.

*“*……Apart from the first few days when I felt obvious pain after returning home, it seemed lighter afterward, basically bearable.” (Participant 17, male, 28‐year‐old)

However, seven participants (20%) mentioned that the pain symptoms they experienced were extremely unpleasant.

*“*……Getting up was difficult after lunch at noon, and semi‐reclining to sleep. There was a feeling of muscle pulling and pain from the thigh to the waist below the wound. The pain was severe, similar to a cramp‐like pain, making me feel so painful that I wanted to die……” (Participant 22, female, 63‐year‐old)

#### 3.1.3 Catastrophizing of pain

Seven participants (20%) were found to focus on and magnify their pain sensations. Manifest in exaggerating the degree of harm caused by the pain and feeling helpless and ruminating over recurrent pain. This high level of attention and magnification can lead to an increase in the severity of pain and emotional distress and a decline in physical function. This phenomenon, often called “catastrophizing,” is a risk factor for chronic pain and may lead to adverse pain‐related outcomes.

“Sometimes, when it hurts, I feel like my pain score could go up to 12 (on a 0‐10 numerical rating scale). That is how much it hurts…… a painful experience I have never had before....” (Participant 19, female, 55‐year‐old)

'I have had a follow‐up examination, and the doctor said I am recovering very well but still in pain. Every time it hurts, I cannot help but think, have my cancer cells metastasized? I want to get a full‐body check‐up....' (Participant 9, female, 67‐year‐old)

#### 3.1.4 Adverse effects caused by pain

Over half of the participants (51.4%) said, in some instances, these responses are bidirectional with pain. Continuous pain may lead to other physical or psychological adverse reactions, and these negative effects may intensify the pain. Such a feedback loop of pain and response might lead to a vicious cycle, making the pain and other symptoms hard to alleviate.

*“*When I was discharged, the doctor told me to cough and do more deep breathing exercises. However, every time I coughed, the wound and my lungs would hurt badly. So I ended up in a vicious cycle where I would be afraid to cough because it hurts, and my lung function has not been recovering well.” (Participant 32, female, 63‐year‐old)

As described by nine participants (25.7%), pain is also one of the leading causes of sleep disturbance.

*“*……One night, I had pain in my abdomen and back. It was very uncomfortable, and I was in a cold sweat due to the pain. I could not sleep and had to get up in the middle of the night to take two painkillers, and it took a long time for me to fall asleep barely. This situation has happened several times, which has made me mentally exhausted for the next few days.” (Participant 29, female, 67‐year‐old)

Twelve participants (34.3%) said they felt overwhelmed by the physical burden and felt that they had become a ‘vulnerable group’ of society because of the pain, which may further increase their psychological burden. It also increases their obstacles to return to daily life, including work, family affairs, and social activities.

*“*Actually, I am not that afraid of pain. However, after I was discharged and went home, my family still treated me completely like a patient, not allowing me to do anything. That made me feel like I was a marginalized and vulnerable group.” (Participant 11, female, 55‐year‐old)

'I used to go shopping with my girlfriends often before the surgery, but after the surgery, I am afraid to walk too long. I am afraid of experiencing pain when I am with them, which would embarrass me.' (Participant 15, female, 35‐year‐old)

'I had to extend my sick leave to my boss from the planned 1 month because I still feel pain and cannot work normally.” (Participant 28, male, 38‐year‐old)

### 3.2 Coping styles for pain

When pain occurs, the patients generally exhibited positive and negative attitudes toward coping. Twenty‐nine participants (82.6%) who coped positively with pain may effectively control it through self‐management and seeking help, thereby accelerating their recovery. Those with a negative attitude may need more support to help them learn to cope with pain more effectively.

#### 3.2.1 Positive coping style

Twenty‐nine participants (82.6%) tended to cope with pain with a positive attitude. When pain occurs, they try to acquire relevant knowledge through various channels and use different methods to relieve it, including medications, nonpharmacological therapies, or even psychological intervention methods.

*“*I have also searched for some information on the Internet. Knowing that this pain is a situation that most people will experience after surgery, I am not so worried.' (Participant 1, female, 38‐year‐old)

'When I was discharged, the doctor prescribed SiWeiPu (Compound Codeine Phosphate And Ibuprofen Sustained Release Tablets sustained‐release tablets). Every time I had pain, I would take medicine according to the instructions. After finishing the medicine, I came to the hospital specifically to get some more painkillers in advance.' (Participant 24, male, 44‐year‐old)

'Besides taking painkillers, I have also tried many methods to relieve my pain, such as acupuncture. I think the effect is excellent.” (Participant 18, female, 58‐year‐old)

#### 3.2.2 Negative coping style

Compared to positive coping with pain, six participants (17.1%) had a slightly pessimistic attitude toward pain. They may consider postoperative pain a ‘normal phenomenon’ and try to endure it as much as possible, even though this might further affect their physical and mental health.

*“*Who does not hurt after surgery? It's all normal, I usually ignore it, and it will pass if I endure it.' (Participant 17, male, 28‐year‐old)

'I do not want others to think I am fragile. Maybe if I do not think about it, its impact on me will be less.” (Participant 27, female, 56‐year‐old)

Alternatively, they might tend to ignore the presence of pain and avoid related activities.

*“*I am also helpless. At first, I used painkillers when I started to feel pain, but they did not work. Later, I just ignored it.' (Participant 13, female, 55‐year‐old)

'As long as I do not move, I will not feel pain, so sometimes I sit or lie down all day long....” (Participant 33, male, 67‐year‐old)

### 3.3 Unmet needs for pain

Pain is one of the most common symptoms of lung cancer patients after surgery.. However, there are unmet needs for patients after discharge regarding knowledge about pain, more individualized pain intervention measures, and continuous pain management.

#### 3.3.1 Lack of pain‐related knowledge

Pain‐related knowledge deficiency was a common issue among 26 participants (72.3%), manifested in their understanding of the fundamental mechanisms of pain, the use of analgesics, and the impact of pain.

Seventeen participants (48.6%) were unsure about the cause of their postoperative pain, notably why it continued even after the wound had healed.

*“*For what reason does this postoperative pain occur? The wound should have completely healed a month after surgery, so why do I still experience occasional pain? Will it impact my future normal life?’ (Participant 11, female, 55‐year‐old)

There was also a widespread misconception about the use of painkillers. Eleven participants (31.4%) feared becoming dependent on them, often equating their use to substance addiction.

“Can I keep taking painkillers? A friend told me that painkillers are like drugs, and it is easy to get addicted. I am apprehensive.” (Participant 12, female, 65‐year‐old)

In addition, due to misleading information, five participants (14.3%) misinterpret their pain as a sign of cancer progression or metastasis, leading to increased anxiety and pain.

*“*However, I saw online that some people say pain might indicate the spread of cancer cells. Who should I believe? If it is true, I am doomed.”(Participant 28, male, 38‐year‐old)

#### 3.3.2 Diversified intervention measures

Almost all of the participants (94.3%) felt frustrated by the limited options beyond painkillers and were not fully satisfied with their consistently effective. Twenty‐four participants (68.6%) expressed the need for more diversified and individualized pain management strategies.

*“*The doctor keeps telling me to use painkillers, but the effect is not very good. Are not there any other methods?” (Participant 26, male, 48‐year‐old)

Eight participants (22.9%) also mentioned they were benefited alternative therapies like acupuncture, part of Traditional Chinese Medicine (TCM), and hoped these methods could be promoted to others suffering from postoperative pain.

*“*Acupuncture really helped alleviate my pain. Our traditional Chinese medical treatments are very impressive. I hope these methods can be promoted to everyone suffering from postoperative pain.” (Participant 18, female, 58‐year‐old)

#### 3.3.3 Continuation of pain management

All participants (100%) expressed a desire for more accessible pain management support after discharge and over half of them (51.4%) hoped that this role should ideally be taken on by authoritative medical personnel. They want more attention, both in the hospital and after discharge.

*“*I wish I could stay in the hospital for a few more days because I feel more cared for when I am there. After being discharged, I am left alone to bear the pain that arises.” (Participant 20, female, 72‐year‐old)

Eighteen participants (51.4%) voiced the need for consistent contact with a professional healthcare provider, expressing that sometimes just receiving reassurances can make them feel better.

*“*I feel that sometimes all I need is reassurance, but I do not have anyone to confide in. It would be great if a professional healthcare provider could keep in touch regularly.” (Participant 28, male, 38‐year‐old)

## 4 DISCUSSION

We identified the thematic framework of pain experiences in home recovery after lung cancer surgery, including three themes: Perception and impact of pain, coping styles for pain, and unmet needs for pain. Studies have shown that the five‐year survival rate of patients after lobectomy for early lung cancer can reach 91.1% and even 94.3% after lung segment resection.^28^ Understanding and improving patients' quality of life after surgery is a critical issue closely related to recovery. The symptoms burden, mainly pain, is an essential factor affecting patients' quality of life and postoperative recovery.^29^ We investigated the pain experiences of lung patients during the home recovery period after surgery, which fills the gap in the existing literature and is one of the main contributions of this study.

This study found that after lung cancer surgery, patients will continue to perceive pain repeatedly, characterized by unpredictability and differences in pain. The chest is densely innervated. Therefore, even minimally invasive incisions under thoracoscopy may exert a certain pressure on or cause damage to the thoracic nerves, thereby causing characteristic intercostal neuralgia.^30^ In addition to the chest, pain can happen at the incision site or in the waist and shoulder area. It often occurs in coexistence in two or more anatomical locations.^31^ Research also indicates that within the framework of health‐related quality of life after lobectomy for lung cancer, 96% of patients express that pain is the symptom they are the most concerned issue.^29^ Our study's results also corroborate this viewpoint.

In our study, patients demonstrated varying degrees of pain perception, with some possibly being more sensitive to pain. In contrast, others may have a higher pain tolerance. It aligns with the biopsychosocial model of pain, which proposes that pain results from the interaction of biological, psychological, and social factors.^32^ Healthcare providers should support patients in forming a holistic understanding of postoperative lung pain from a biopsychosocial perspective. Research has illustrated that catastrophizing about pain can lead to enhanced pain perception, inappropriate use of pain coping strategies, and limitations in physical and psychological functions related to pain.^33^, ^34^ Therefore, pain management strategies may need to include psychological interventions to reduce pain catastrophizing. It may involve cognitive behavioral techniques and the involvement of mental health professionals in their care.^35^ In addition, the findings also reveal that the continuous perception of pain causes physiological harm to patients, exacerbates their psychological burden, and leads to absences or delays in family affairs, work, and social activities. It is consistent with the findings of Mody et al., who found through surveys that pain is the most common and longest‐lasting symptom after lung cancer surgery and is associated with decreased activity levels.^36^ It also means that while focusing on the pain in patients, the additional burdens caused by it also need to be assessed and intervened to promote the holistic recovery of the “physical‐psychological‐social” attributes.

Coping style is a vital concept in the symptom management of cancer patients. It is a conscious psychological process to manage stressful life stressors, such as pain, sleep, depression, and psychological distress.^37^, ^38^, ^39^, ^40^ Typical coping styles include a positive tendency to be adaptive in stressful situations and a negative tendency to be adaptive in the same stressful situation.^41^ High levels of positivity may give patients more expectations and confidence in coping with adverse challenges.^42^, ^43^ In this study, most patients adopt positive coping styles when pain emerges, including obtaining relevant knowledge and actively trying different interventions to relieve it. Generally speaking, an upbeat coping style helps to reduce harmful emotions and psychological distress under pressure and improve the quality of life.^44^, ^45^ We discovered that some patients have a slightly pessimistic attitude toward pain, unwilling to face or give up. There are many possible reasons. It may associate with the continuous occurrence of pain, the failure of intervention, and the lack of relevant knowledge mentioned in this study. Besides, under the influence of traditional Confucianism, the subconscious of the Chinese is cultivated into a resolute and brave character, which may show a pronounced forbearance tendency toward pain.^46^, ^47^ It explains the results of negative coping of patients from another angle. However, negative coping harms pain management. Studies have authenticated that pain tolerance and non‐treatment will lead to pain symptoms not being discovered in time, thus aggravating the impact of symptoms and reducing patients' quality of life.^48^, ^49^ Therefore, when developing pain management programs, medical staff should fully assess the patient's coping style and strive to promote positive, constructive, problem‐oriented coping strategies.

This study indicated that discharged patients lacked knowledge of pain and were eager for health education. Due to the practice and development of enhanced recovery after thoracic surgery, hospital stay is becoming shorter,^50^ so the time of contact with medical staff is reduced, which may also lead to decreased patients ‘opportunities to receive pain education.^51^ The application of planned health education can influence postoperative pain, state anxiety, FEV 1, and FEF25/75 values of lung cancer to positively impact the clinical recovery process.^52^ The survey also pointed to the need for higher‐quality pain education for patients in the future.^53^ Therefore, various educational strategies can be employed to meet their pain knowledge needs. In this study, the patients mentioned that they wanted to accept more diversified analgesic measures besides drug intervention, among which the TCM therapy was a high‐frequency way, such as herbal medicine and acupuncture. A study on patients attitudes toward pain found that patients had limited knowledge of pain medication and preferred combining medication with nonpharmacological and complementary therapies.^54^ Non‐pharmacologic therapies such as acupressure, acupuncture, music therapy, and mindfulness meditation have also been demonstrated to be effective in relieving pain,^55^, ^56^, ^57^ so these alternative therapies can be used as an adjunct to patients who need individualized pain intervention or who are not responding well to medication. We also discovered that patients urgently need pain management based on the professional continuity of medical staff after discharge. Providing pain management information to patients during home recovery at multiple points is beneficial.^58^ With the rapid development of Internet technology in the medical field, remote pain management solutions based on digital therapy are expected to become better actions.^59^, ^60^ Digital therapy combines scientific and medical knowledge to provide innovative ways to assess, treat and manage pain. Its effectiveness has been confirmed.^61^, ^62^, ^63^, ^64^ In the future, when providing pain management for patients in the home recovery period after lung surgery, interventions supported by higher‐level evidence should be comprehensively considered to meet their needs with improve their quality of life and satisfaction with medical treatment.

## 5 LIMITATIONS

Firstly, although we used maximum variance sampling to recruit patients to mitigate a potential lack of data richness, our results should be treated with caution when extrapolating because our participants were from the single center. Differences in surgeon performance and quality of perioperative care at different hospitals may contribute to changes in pain perception after discharge. Secondly, we focused on the pain experience of postoperative lung cancer patients. However, whether patients received chemotherapy, immunotherapy, or targeted therapy after surgery was not considered, factors may also cause a pain burden on patients, which may cause a lack of information for constructing the experience framework. Future studies can consider more elements to build a more comprehensive pain‐related complex relationship framework. Finally, most participants described their experiences as recollective, which may lead to recall bias in the data. In the upcoming studies, we intend to incorporate both quantitative and qualitative data for triangulation analysis to gain deeper insights, and to mitigate recall bias through long‐term multiple return visits.

## 6 CONCLUSIONS

This study sheds light on the complexity of the pain experience of lung cancer patients during their home recovery after surgery, as well as their coping strategies and currently unmet management needs. These findings may have a potential reference value for medical staff to develop individualized pain management programs and improve the quality of care.

## AUTHOR CONTRIBUTIONS

**Cheng Lei:** Conceptualization (equal); data curation (equal); formal analysis (equal); funding acquisition (equal); methodology (equal); software (equal); visualization (equal); writing – original draft (equal); writing – review and editing (equal). **Ruoyan Gong:** Conceptualization (equal); data curation (equal); formal analysis (equal); validation (equal); visualization (equal); writing – original draft (equal). **Jiayuan Zhang:** Conceptualization (equal); writing – original draft (equal). **Kejimu Sunzi:** Methodology (equal); visualization (equal); writing – original draft (equal). **Na Xu:** Data curation (equal); resources (equal); writing – original draft (equal). **Qiuling Shi:** Conceptualization (equal); funding acquisition (equal); project administration (equal); resources (equal); supervision (equal); writing – original draft (equal); writing – review and editing (equal).

## FUNDING INFORMATION

This research was funded by The Chongqing Postgraduate Scientific Research Innovation Project (CYB22220) and The National Key Research and Development Program of the Ministry of Science and Technology of China‐Intergovernmental International Cooperation Project on Science and Technology Innovation (2022YFE0133100).

## CONFLICT OF INTEREST STATEMENT

The authors declare no conflict of interest.

## Supporting information


**Appendix S1:** Supporting Information

## Data Availability

The dataset used and analyzed during the current study is not publicly available due to the sensitivity of the data (transcribed interviews from patients). It may be available from the corresponding author upon reasonable request.
