# Supplementary material for: Pain experience of lung cancer patients during home recovery after surgery: A qualitative descriptive study
Source: Cancer Med. 2023 Oct 3;12(19):20212–23. doi: 10.1002/cam4.6616 (PMC10587973; doi:10.1002/cam4.6616)
Supplement: Supplementary file 1 — Appendix S1: Supporting Information [file CAM4-12-20212-s001.docx]

**S1. Outline of the interview.**

| **Question** | **Prompts** |
| --- | --- |
| 1.Please describe the experience of pain during recovery at home after surgery. | Such as duration, location... |
| 2.What is your perspective on the phenomenon of pain? | Such as attitude... |
| 3.How has the experience of pain affected you? | Not limited to the pain itself... |
| 4. Please describe how you dealt with the pain when it happened. | Such as the strategies adopted... |
| 5. What do you think of the process of managing pain? |  |
| 6.Do you have any particular needs or suggestions for managing pain? |  |
| 7.Is there any additional information you would like to provide? |  |

**S2. Detailed phases and description in themes analysis.**

| **Phases** | **Description** |
| --- | --- |
| 1.Familiarising yourself with your data | Two researchers* familiarized with data, in order to achieve the purpose of "immersing" in the data by reading through all the participants' transcript texts several times. |
| 2. Generating initial codes | Based on the reading and familiarity with the data, an initial list of ideas about the pain experience contained in the data and what is interesting about it is generated. This phase generated the initial codes from the data, which can be the words of the participants or the summarized from the data. |
| 3. Searching for themes | At the beginning of phase 3, all the data is initially encoded and collated, and a long list of different codes are identified throughout the data set. This phase will focus on analyzing the broader theme level, classifying different code into potential themes, and collating all relevant encoded data excerpts within the identified themes. |
| 4. Reviewing themes | Checking in the themes work in relation to the coded extracts (Level 1) and the entire data set (Level 2), generating a thematic map of the analysis. |
| 5. defining and naming themes | Ongoing analysis to refine the specifics of each theme, and the overall story the analysis tells; generating clear definitions and names for each theme. |
| 6. Producing the report | The final opportunity for analysis. Selection of vivid, compelling extract examples, final analysis of selected extracts, relating back of the analysis to the research question and literature, producing a scholarly report of the analysis. |

*Two researchers are CL and RG.

**S3. Research themes, sub-themes and relative quotes.**

| Themes | Sub-themes | Quotes |
| --- | --- | --- |
| *Perception and Impact of Pain* | *Unpredictability of pain* | - *'I have been discharged for two weeks, and the wound is still excruciating. How long will it take to end?' (Participant 3, male, 57-year-old)* - *'It has been almost two months since my surgery, and suddenly yesterday afternoon, I started feeling a sharp pain in my chest, like a needle prick, which lasted through the night.' (Participant 12, female, 65-year-old)* - *'Last week, I suddenly felt a pulling pain in my back. It took half a day to relieve a bit, but the painkillers seemed ineffective.' (Participant 13, female, 55-year-old)* - *'When I was just discharged, the pain was severe. It was not until around a month later that it started to ease. However, recently, there have been bouts of pain from time to time. It has been almost a year, and I cannot figure out why it still hurts!' (Participant 23, male, 62-year-old)* - *'Oh my, I did not expect this. Since I had my lobectomy, the pain has been unbearable. It is much worse than I originally thought. I feel so uncomfortable every day. I hope this situation improves soon.' (Participant 8, female, 52-year-old)* - *'...especially at the surgical incision, it is still very painful...'.(Participant 7, male, 55-year-old)* - *'I feel a pulling pain in the shoulder area of the surgery...' (Participant15, female, 48-year-old)* - *'I have been home for two weeks now, and there is still a pain in my chest.' (Participant 23, male, 52-year-old)* - *'There were a few times when my arms hurt so much I could not lift it. Is this normal?' (Participant18, female, 48-year-old)* |
|  | *Different levels of pain severity* | - *'……Apart from the first few days when I felt obvious pain after returning home, it seemed lighter afterward, basically bearable.' (Participant 17, male, 28-year-old)* - *'……Getting up was difficult after lunch at noon, and semi-reclining to sleep. There was a feeling of muscle pulling and pain from the thigh to the waist below the wound. The pain was severe, similar to a cramp-like pain, making me feel so painful that I wanted to die……' (Participant 22, female, 63-year-old)* |
|  | *Catastrophizing of pain* | - *'Sometimes, when it hurts, I feel like my pain score could go up to 12 (on a 0-10 numerical rating scale). That is how much it hurts…... a painful experience I have never had before....' (Participant 19, female, 55-year-old)* - *'I have had a follow-up examination, and the doctor said I am recovering very well but still in pain. Every time it hurts, I cannot help but think, have my cancer cells metastasized? I want to get a full-body check-up....' (Participant 9, female, 67-year-old)* |
|  | *Adverse effects caused by pain* | - *'When I was discharged, the doctor told me to cough and do more deep breathing exercises. However, every time I coughed, the wound and my lungs would hurt badly. So I ended up in a vicious cycle where I would be afraid to cough because it hurts, and my lung function has not been recovering well.' (Participant 32, female, 63-year-old)* - *'……One night, I had pain in my abdomen and back. It was very uncomfortable, and I was in a cold sweat due to the pain. I could not sleep and had to get up in the middle of the night to take two painkillers, and it took a long time for me to fall asleep barely. This situation has happened several times, which has made me mentally exhausted for the next few days.' (Participant 29, female, 67-year-old)* - *'Actually, I am not that afraid of pain. However, after I was discharged and went home, my family still treated me completely like a patient, not allowing me to do anything. That made me feel like I was a marginalized and vulnerable group.' (Participant 11, female, 55-year-old)* - *'I used to go shopping with my girlfriends often before the surgery, but after the surgery, I am afraid to walk too long. I am afraid of experiencing pain when I am with them, which would embarrass me.' (Participant 15, female, 35-year-old)* - *'I had to extend my sick leave to my boss from the planned one month because I still feel pain and cannot work normally.' (Participant 28, male, 38-year-old)* |
| *Coping Styles for Pain* | *Positive coping style* | - *'I have also searched for some information on the Internet. Knowing that this pain is a situation that most people will experience after surgery, I am not so worried.' (Participant 1, female, 38-year-old)* - *'When I was discharged, the doctor prescribed SiWeiPu (Compound Codeine Phosphate And Ibuprofen Sustained Release Tablets sustained-release tablets). Every time I had pain, I would take medicine according to the instructions. After finishing the medicine, I came to the hospital specifically to get some more painkillers in advance.' (Participant 24, male, 44-year-old)* - *'Besides taking painkillers, I have also tried many methods to relieve my pain, such as acupuncture. I think the effect is excellent.' (Participant 18, female, 58-year-old)* |
|  | *Negative coping style* | - *'Who doesn't hurt after surgery? It's all normal, I usually ignore it, and it will pass if I endure it.' (Participant 17, male, 28-year-old)* - *'I don't want others to think I am fragile. Maybe if I don't think about it, its impact on me will be less.' (Participant 27, female, 56-year-old)* - *'I am also helpless. At first, I used painkillers when I started to feel pain, but they didn't work. Later, I just ignored it.' (Participant 13, female, 55-year-old)* - *'As long as I don't move, I won't feel pain, so sometimes I sit or lie down all day long....' (Participant 33, male, 67-year-old)* |
| *Unmet Needs for Pain* | Lack of pain-related knowledge | - *'For what reason does this postoperative pain occur? The wound should have completely healed a month after surgery, so why do I still experience occasional pain? Will it impact my future normal life?' (Participant 11, female, 55-year-old)* - *'Can I keep taking painkillers? A friend told me that painkillers are like drugs, and it is easy to get addicted. I am apprehensive.' (Participant 12, female, 65-year-old)* - *'However, I saw online that some people say pain might indicate the spread of cancer cells. Who should I believe? If it is true, I am doomed.' (Participant 28, male, 38-year-old)* |
|  | Diversified intervention measures | - *'The doctor keeps telling me to use painkillers, but the effect is not very good. Aren't there any other methods?' (Participant 26, male, 48-year-old)* - *'Acupuncture really helped alleviate my pain. Our traditional Chinese medical treatments are very impressive. I hope these methods can be promoted to everyone suffering from postoperative pain.' (Participant 18, female, 58-year-old)* |
|  | Continuation of pain management | - *'I wish I could stay in the hospital for a few more days because I feel more cared for when I am there. After being discharged, I am left alone to bear the pain that arises.' (Participant 20, female, 72-year-old)* - *'I feel that sometimes all I need is reassurance, but I don't have anyone to confide in. It would be great if a professional healthcare provider could keep in touch regularly.' (Participant 28, male, 38-year-old)* |
